# The attributable mortality of sepsis for acute kidney injury: a propensity-matched analysis based on multicenter prospective cohort study

**DOI:** 10.1080/0886022X.2022.2162415

**Published:** 2023-01-13

**Authors:** Hui-Miao Jia, Yi-Jia Jiang, Xi Zheng, Wen Li, Mei-Ping Wang, Xiu-Ming Xi, Wen-Xiong Li

**Affiliations:** a Department of Surgical Intensive Critical Unit, Beijing Chao-yang Hospital, Capital Medical University, Beijing, China; b Department of Critical Care Medicine, Fuxing Hospital, Capital Medical University, Beijing, China; c Department of Epidemiology and Health Statistics, School of Public Health, Capital Medical University, Beijing, China

**Keywords:** Sepsis, acute kidney injury, attributable mortality

## Abstract

**Background:**

Both sepsis and AKI are diseases of major concern in intensive care unit (ICU). This study aimed to evaluate the excess mortality attributable to sepsis for acute kidney injury (AKI).

**Methods:**

A propensity score-matched analysis on a multicenter prospective cohort study in 18 Chinese ICUs was performed. Propensity score was sequentially conducted to match AKI patients with and without sepsis on day 1, day 2, and day 3–5. The primary outcome was hospital death of AKI patients.

**Results:**

A total of 2008 AKI patients (40.9%) were eligible for the study. Of the 1010 AKI patients with sepsis, 619 (61.3%) were matched to 619 AKI patients in whom sepsis did not develop during the screening period of the study. The hospital mortality rate of matched AKI patients with sepsis was 205 of 619 (33.1%) compared with 150 of 619 (24.0%) for their matched AKI controls without sepsis (*p* = 0.001). The attributable mortality of total sepsis for AKI patients was 9.1% (95% CI: 4.8–13.3%). Of the matched patients with sepsis, 328 (53.0%) diagnosed septic shock. The attributable mortality of septic shock for AKI was 16.2% (95% CI: 11.3–20.8%, *p* < 0.001). Further, the attributable mortality of sepsis for AKI was 1.4% (95% CI: 4.1–5.9%, *p* = 0.825).

**Conclusions:**

The attributable hospital mortality of total sepsis for AKI were 9.1%. Septic shock contributes to major excess mortality rate for AKI than sepsis.

**Registration for the multicenter prospective cohort study:**

registration number ChiCTR-ECH-13003934

## Introduction

The rising prevalence and mortality of sepsis and acute kidney injury (AKI) are main threats to the survival of critically ill patients worldwide. Both sepsis and AKI are diseases of major concern in intensive care unit (ICU) [1–3]. In critically ill patients, a variety of factors can cause AKI [4], but sepsis is the most common trigger of AKI [5]. Approximately, 40–50% of cases of the development of AKI is associated with sepsis [6]. Septic AKI is distinct from nonseptic AKI, with differences in pathogenesis, clinical characteristics and outcomes [7–9]. Limited understanding of pathophysiologic mechanisms has prevented the evolution of effective therapies of sepsis and AKI. The mortality rate of septic AKI is up to 30–60%, depending on severity of illness [6,10]. Up to now, no study assessed the accurate contribution of sepsis for mortality of AKI patients. It is assumed that whether sepsis develops before, simultaneously or after AKI, it would contribute excess mortality for AKI. Therefore, we conducted the propensity score-matched analysis between AKI patients with and without sepsis to evaluate the attributable mortality of sepsis for AKI patients.

## Materials and methods

### Study setting and population

This is a retrospective propensity score-matched analysis based on database of a prospective cohort study about sepsis epidemiology sponsored by China Critical Care Sepsis Trial (CCCST) workgroup, which was performed in 18 Chinese trial sites of 16 hospitals between January 1, 2014, and August 31, 2015. The database included adult patients of 4910 who stayed longer than 24 h in ICU. We screened and included patients who diagnosed AKI in the 4910 patients within 5 days after ICU admission. Then, we excluded patients from the AKI patients. The exclusion criteria included (1) operated with nephrectomy or kidney transplantation; (2) acquired insufficient data. A complete list of trial sites is provided in the Supplementary File. The protocol of study was registered on August 3, 2013. The study was approved in all participating ICUs by their Hospital Human Ethics Committee. The chief ethics number was 2013FXHEC-KY2018. The registration number was ChiCTR-ECH-13003934. A preprint of the manuscript was online. An article about the risk factors, clinical features and outcome of new-onset AKI on this database was previously published. Now we focused AKI with sepsis and explored the attributable mortality of sepsis for AKI patients [11]. Informed consent from patients or their next of kin was obtained before patients joined in the study. All enrolled patients adhere to the following management principles: active treatment of primary disease and complications; and the same principles of treatment with antibiotics, nutritional metabolism and organ support.

### Clinical endpoint and definitions

The primary endpoint was hospital mortality of AKI patients. The diagnosis of sepsis and septic shock was according to the sepsis 3.0 definition of the American College of Chest Physicians/Society of Critical Care Medicine criteria [12]. The definitions of AKI and AKI classification were depended on the serum creatinine and urine output criteria proposed by Kidney Disease: Improving Global Outcomes (KDIGO) [13]. AKI with sepsis is defined as patients who develop AKI and sepsis, whether sepsis develops before, simultaneously or after AKI diagnosis. In this study, we focused AKI and sepsis diagnosed in 5 days after ICU admission. Renal replacement therapy (RRT) was initiated according to medical necessity of patients and decision of the treating clinician. The baseline creatinine was defined as follows: if at least five values were available the median of all values available from six months to six days prior to enrollment was used. Otherwise, the lowest value in the five days prior to enrollment was used. If no pre-enrollment creatinine was available or the emergency patient’s serum creatinine was abnormal at the time of admission, the baseline creatinine was estimated using the Modification of Diet in Renal Disease (MDRD) equation assuming that baseline eGFR is 75 mL/min per 1.73 m^2^ [14].

### Data collection

The information collected included demographic characteristics, chronic illnesses, diagnosis, pre-ICU medications and treatment (whether or not used nephrotoxic drugs, nephrotoxic drugs included angiotensin converting enzyme inhibitors, non-steroidal anti-inflammatory drug, Amikacin and Amphotericin B), the reason for ICU admission, acute physiology and chronic health evaluation (APACHE II) on the day of ICU admission, baseline serum creatinine, creatinine values every 12 h and hourly urine output on ICU admission and thereafter until transferred out of ICU, use of mechanical ventilation, as well as serum lactate, use of vasoactive drugs, sequential organ failure assessment (SOFA) score every day in the first 7 days after ICU admission. We also collected time of diagnosing sepsis, septic shock and AKI after ICU admission, AKI classification, ICU stay, hospital stay, hospital mortality and 30-day mortality.

### Propensity score matching

**Figure 1.**
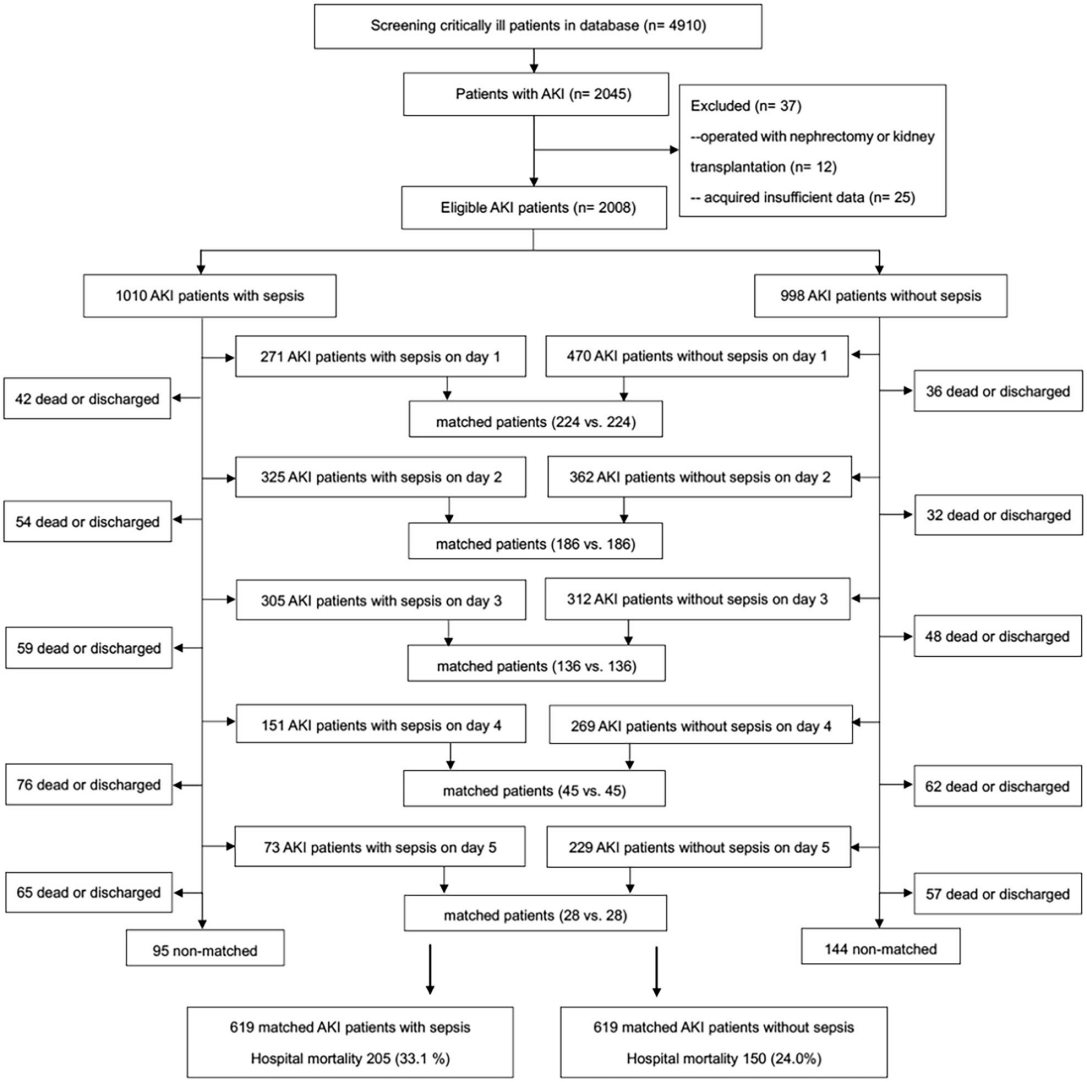
Study flow diagram. The propensity score for AKI with sepsis on the first ICU day (day 1) included patients diagnosed AKI and sepsis on day 1. Excluding patients who were matched on day 1, discharged and dead on day 1, patients of AKI with sepsis on the second ICU day (day 2) were identified in the rest of patients, including patients who diagnosed AKI on day 1 with renal recovery on day 2. Sequentially, excluding patients who were matched on day 2, discharged and dead on day 2, patients of AKI with sepsis on the third ICU day (day 3) were identified, including patients who diagnosed AKI on day 1 or day 2 but with renal recovery on day 3. Patients of AKI with sepsis on days 4-5 were constructed correspondingly. Further, cohort of AKI without sepsis on the first ICU day included patients diagnosed AKI on day 1 who never developed sepsis during the study screening period. Excluding patients who were matched on day 1, discharged and dead on day 1, patients of AKI who never developed sepsis during the study screening time on the second ICU day were identified in the rest of patients, including patients who diagnosed AKI on day 1 with renal recovery on day 2. Sequentially, excluding patients who were matched on day 2, discharged and dead on day 2, patients of AKI who never developed sepsis during the study screening time on the third ICU day were identified, including patients who diagnosed AKI on day 1 or day 2 but with renal recovery on day 3. Patients of AKI without sepsis on days 4-5 were constructed correspondingly. *AKI* acute kidney injury, *ICU* intensive care unit.

A patient in the exposure group with is matched with a comparable patient who has the most similar propensity score. Propensity score matching was constructed for AKI patients with and without sepsis (1:1) at five different time points: day 1, day 2, day 3, day 4 and day 5 after ICU admission (Figure 1). The propensity score based on baseline characteristics and clinical covariates was used to adjust the differences in matched patients with and without sepsis, which was constructed depending on logistic regression and including variables of age, sex, body mass index (BMI), chronic illnesses (chronic obstructive pulmonary disease (COPD) or asthma, cardiovascular disease, chronic liver disease, cancer, diabetes, hypertension, chronic kidney disease (CKD)), AKI classification, nonrenal SOFA score, and mechanical ventilation. Nonrenal SOFA score was remarkably correlated linearly with APACHE II. AKI patients with sepsis were matched 1:1 with controls of AKI patients without sepsis. Then, the hospital mortality in each matched AKI group was calculated and attributable mortality of total sepsis for AKI was estimated.

Matched AKI patients with sepsis and their controls of AKI patients without sepsis were subgrouped by the severity of sepsis (sepsis, septic shock). The severity of sepsis (sepsis, septic shock) was distinguished according to whether the patients met diagnostic criteria of septic shock on the time of diagnosis. The mortality in each matched subgroup was calculated and attributable mortalities of sepsis and septic shock for AKI were estimated, respectively.

### Statistical analysis

Continuous variables were presented as mean ± standard deviation (SD) or median values (25th and 75th percentiles), categorical variables were presented as percentiles. Continuous data between two groups was compared using the Student’s *t*-test or Mann–Whitney U-tests, and categorical variables used the Chi square test or Fisher’ s exact test. Propensity score was constructed to match AKI patients with and without sepsis. The caliper width was set to 0.1 of the standard deviation of the logit of the propensity score. Covariate balance before and after matching was examined using standardized differences, with values 0.15 considered as evidence of meaningful differences [15]. Pearson or Spearman correlation test was used to estimate the correlation between two variables. The excess mortality of AKI patients attributable to sepsis was calculated by subtracting the mortality of matched AKI patients without sepsis from the mortality of matched AKI patients with sepsis. 95% confidence interval (CI) for the attributable mortality difference was calculated by Newcombe’s method [16]. The McNemar’s test was used for sensitivity analysis to assess the stability of outcomes [15]. The primary endpoint was further evaluated by Kaplan–Meier curves (log-rank test). For all analyses, statistical significance was indicated by two-sided *p* < 0.05. SPSS statistics 22 (IBM, Chicago, IL, USA) and R 2.1.2 were used for statistical analyses.

## Results

A total of 4910 adult critically ill patients were screened. Of them, 2045 developed AKI within 5 days after ICU admission. After excluding 37 ineligible patients, there were 2008 AKI patients finally included in this study. The characteristics of included patients are performed in Table 1. The study flow diagram (Figure 1) illustrates the ICU treatment day on which sepsis was diagnosed and the sequential matching procedure. Supplemental Table 1 shows the characteristics of matched AKI patients with sepsis and their controls without sepsis in five separate time points.

**Table 1.** Characteristics of the whole cohort AKI.

Variables	Total AKI (*n* = 2008)
Demographics	
Age, years	66 (52, 78)
Male gender	1369 (68.2)
BMI, kg/m^2^	19.1 (17.5, 21.1)
Comorbidities	
COPD/asthma	129 (6.4)
Cardiovascular disease	395 (19.7)
Chronic liver disease	60 (2.8)
Cancer	188 (9.4)
Diabetes	416 (20.7)
Hypertension	788 (39.2)
CKD stage 1–2	125 (6.2)
Stage 3	93 (4.6)
Sepsis	1007 (50.1)
Admission type	
Medical	825 (41.1)
Surgical	446 (22.2)
Emergency	737 (36.7)
APACHE II score	19 (13, 25)
AKI stage 1	1193 (59.4)
Stage 2–3	815 (40.6)
Nonrenal SOFA	5 (3, 8)
Use of nephrotoxic drugs	171 (8.5)
Mechanical ventilation	1476 (73.5)
Use of vasopressor	976 (48.6)
Use of RRT	601 (29.9)
RRT modality	
CVVH	548 (27.3)
CVVHD	23 (1.1)
CVVHDF	30 (1.5)
Outcomes	
ICU stay (days)	7 (4, 14)
Hospital stay (days)	17 (10, 26)
30-day mortality	549 (27.3)
Hospital mortality	646 (32.2)

Values are median (interquartile range) or *n* (%), *BMI* body mass index, *APACHE II* acute physiology and chronic health evaluation, *SOFA* sequential organ failure assessment, *COPD* chronic obstructive pulmonary disease, *CKD* chronic kidney disease, *ICU* intensive care unit, *RRT* renal replacement therapy, *CVVH* continuous venovenous hemofilration, *CVVHD* continuous venovenous hemodialysis, *CVVHDF* continuous venovenous hemodiafiltration.

**Figure 2.**
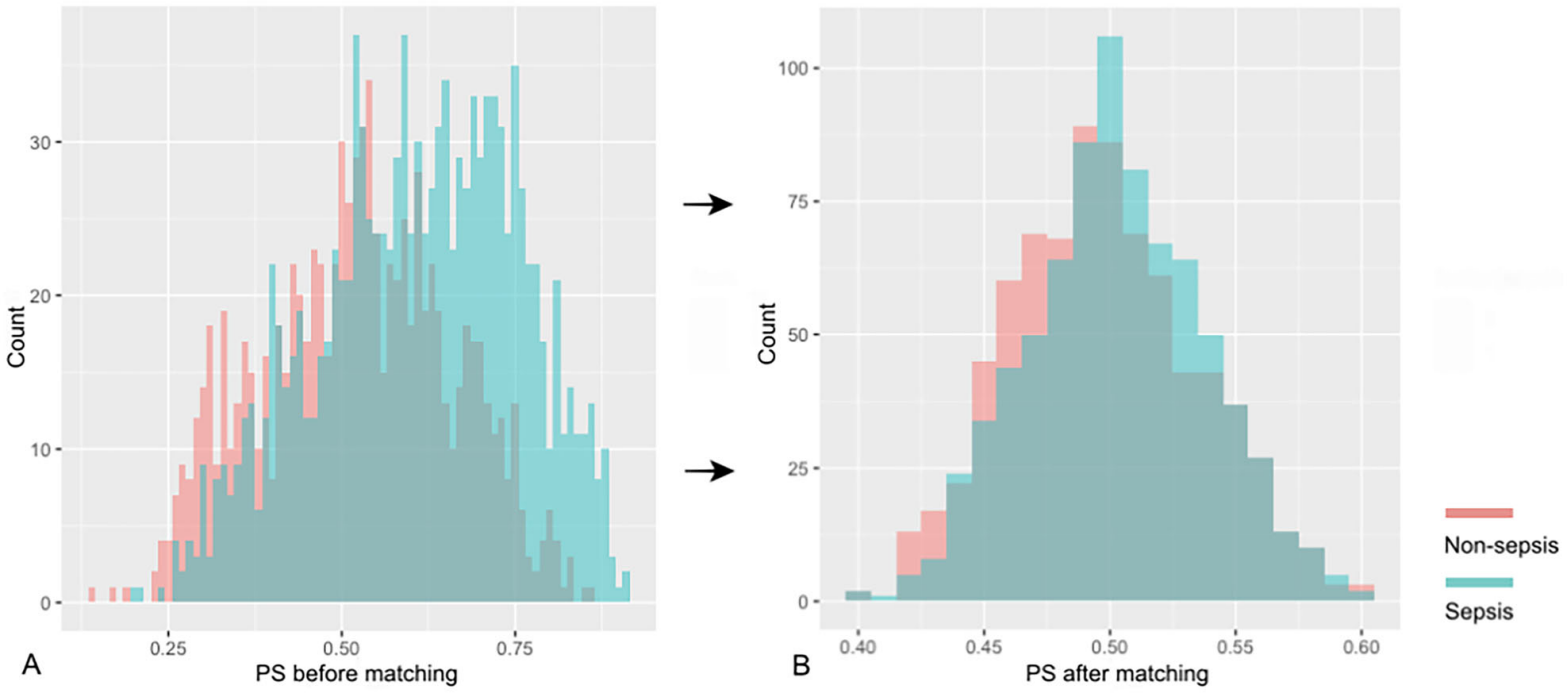
The distributions of propensity score before and after matching in cohorts with and without sepsis: (A) propensity score before matching; (B) propensity score after matching. *PS* propensity score.

**Figure 3.**
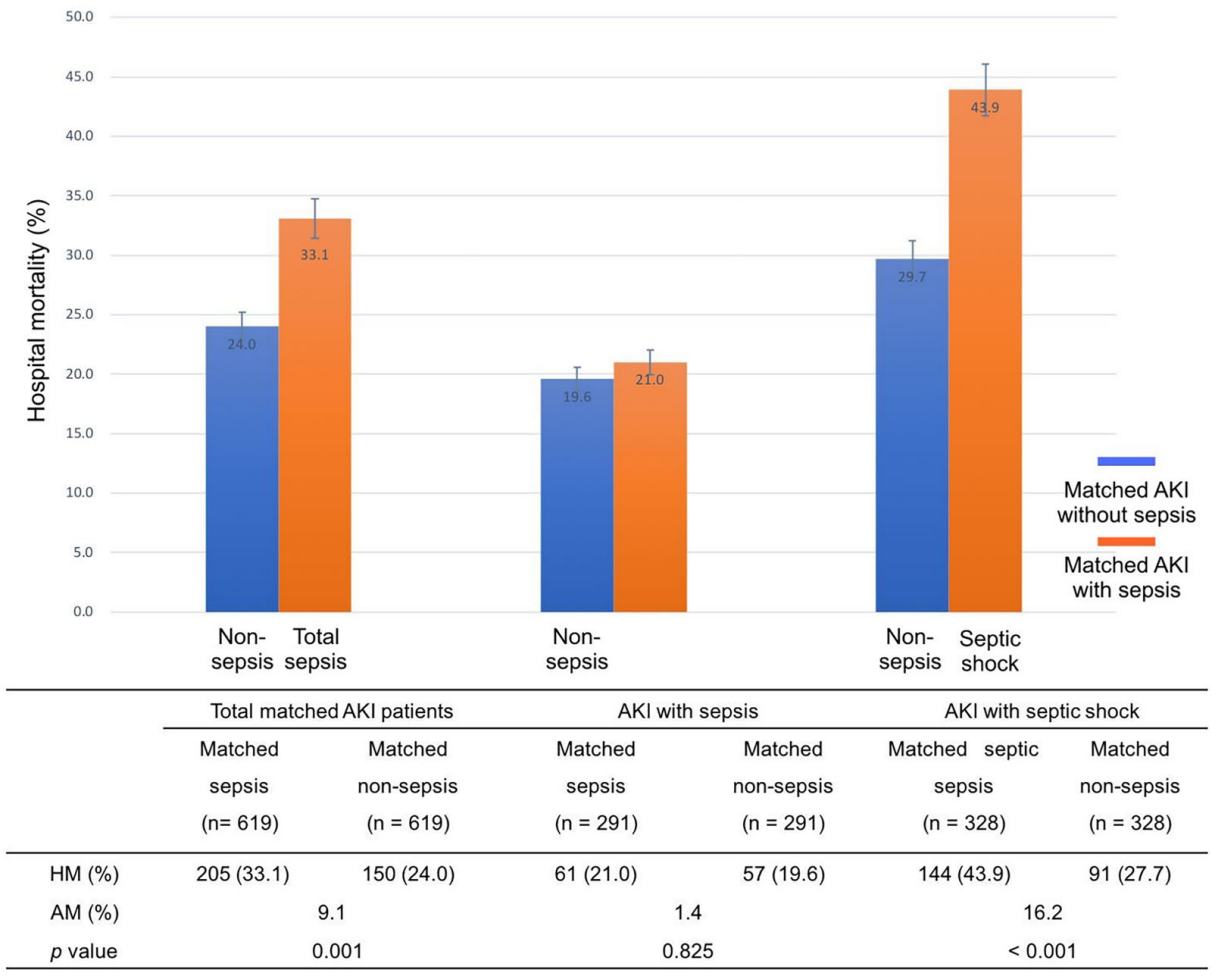
The attributable mortalities of total sepsis and different severity of sepsis (sepsis and septic shock) for AKI. *AKI* acute kidney injury, *AM* attributable mortality, *HM* hospital mortality.

**Figure 4.**
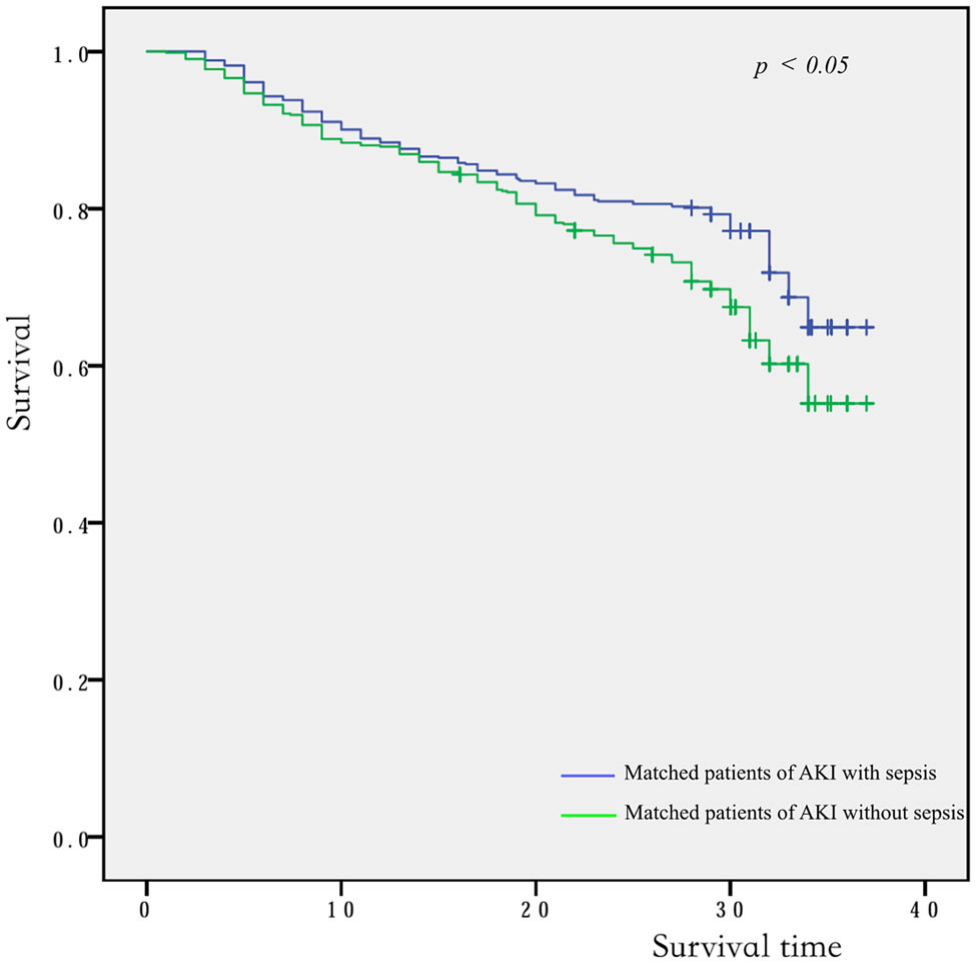
The Kaplan-Meier curves of the two groups. *AKI* acute kidney injury.

Of the 1010 AKI patients with sepsis, 619 (61.3%) were matched to 619 AKI patients in whom sepsis did not develop during the screening period of the study (Figure 1). The distributions of propensity score before and after matching in cohorts with and without sepsis are shown in Figure 2. Patient characteristics of the matched pairs are presented in Table 2. After matching, the groups were balanced regarding the matched variables with small standardized differences (Table 2). RRT was used in 167 (27.0%) and 160 (25.8%) patients in matched AKI patients with and without sepsis, respectively (*p* = 0.699). The length of ICU and hospital stay was 8.0 days (4.0–15.5 d), 17.0 days (11.0–25.0 d) for AKI patients with sepsis and 6.0 days (4.0–11.0 d), 18.0 days (10.0–28.0 d) for the nonsepsis AKI control patients, respectively (*p* = 0.001, *p* = 0.158). The hospital mortality rate of matched AKI patients with sepsis was 205 of 619 (33.1%) compared with 150 of 619 (24.0%) for their matched AKI controls without sepsis (*p* = 0.001). The attributable mortality of total sepsis for AKI patients was 9.1% (95% CI: 4.8–13.3%). The attributable mortalities of total sepsis and different severity of sepsis (sepsis and septic shock) for AKI are shown in Figure 3. The Kaplan-Meier curves and comparison of the distribution of the two groups are shown in Figure 4.

**Table 2.** A comparison of characteristics between matched AKI patients with and without sepsis.

Variables	Matched AKI with sepsis (*n* = 619)	Matched AKI without sepsis (*n* = 619)	*p* value 0.409
Age, years	67 (53, 79)	66 (52, 78)	0.480
Male gender	400 (64.6)	385 (62.2)	0.585
BMI, kg/m^2^	19.1 (17.6, 21.1)	19.4 (17.4, 21.1)	0.888
COPD/asthma	44 (7.1)	33 (5.3)	0.239
Cardiovascular disease	136 (22.0)	117 (18.9)	0.204
Chronic liver disease	13 (2.1)	14 (2.3)	1.000
Cancer	48 (7.8)	59 (9.5)	0.312
Diabetes	154 (24.9)	138 (22.3)	0.315
Hypertension	261 (42.2)	259 (41.8)	0.954
CKD stage 1–2	39 (6.3)	38 (6.1)	0.912
stage 3	30 (4.8)	27 (4.4)	0.834
AKI stage 2–3	331 (53.5)	305 (49.3)	0.629
stage 1	288 (46.5)	314 (51.7)	
Use of nephrotoxic drugs	56 (9.0)	50 (8.1)	0.536
Surgical operation	125 (20.2)	133 (21.8)	0.681
Nonrenal SOFA on matched day	5 (3, 8)	5 (3, 8)	0.528
APACHE II score	18 (12, 23)	18 (13, 23)	0314
Mechanical ventilation	444 (71.7)	454 (73.3)	0.567

Values are median (interquartile range) or *n* (%), *BMI* body mass index, *COPD* chronic obstructive pulmonary disease, *CKD* chronic kidney disease, *AKI* acute kidney injury, *SOFA* sequential organ failure assessment.

Matched AKI patients with and without sepsis were subgrouped according to the severity of sepsis (sepsis, septic shock). Of the matched patients with sepsis, 328 (53.0%) diagnosed septic shock. The mortality rate showed remarkably higher in matched AKI patients with septic shock (43.9%) than their controls of patients without sepsis (27.7%). The attributable mortality of septic shock for AKI was 16.2% (95% CI: 11.3–20.8%, *p* < 0.001). Further, the attributable mortality of sepsis for AKI was 1.4% (95% CI: −4.1–5.9%, *p* = 0.825), although there was no significant difference of mortality rate observed between matched AKI patients with and without sepsis (21.0% vs. 19.6%).

## Discussion

Sepsis and AKI are inextricably common diseases in critically ill patients. Sepsis is a leading cause of AKI, and AKI is a frequent complication of sepsis [4,5]. Many researches’ results convincingly showed the ‘intimate’ bond between sepsis and AKI in ICU patients. For instance, AKI in up to half of septic patients was reported in the BEST Kidney and FINNAKI studies [6,17]. Study by Vaara ST, et al. [18] estimated the attributable mortality of AKI. Kelly F, et al. [19] examined long-term mortality in sepsis patients compared to hospitalized non-sepsis controls. However, none of studies accurately calculated the attributable mortality of sepsis for AKI. For this purpose, this sequentially propensity-matched analysis calculated the attributable mortality of sepsis for AKI. Propensity score balanced the baseline characteristics and clinical covariates of diabetes mellitus, COPD, cardiovascular disease, etc in two groups of AKI patients with and without sepsis. The main findings show that the estimated excess hospital mortality statistically attributable to sepsis for AKI was 9.1 percentage points (95% CI: 4.8–13.3% percentage points). Septic shock contributed to major excess mortality for AKI than sepsis. The findings further strengthen the role of sepsis as a significant leading cause for mortality of AKI.

Sepsis is defined as life-threatening organ dysfunction caused by a dysregulated host response to infection. Abundant releases of inflammatory cytokines and leukocyte activation may result in capillary plugging and micro-thrombi, as well as production of reactive oxygen species and induction of nitric oxide synthase, which further destroy the endothelial barrier and the glycocalyx leading to organ edema and systemic hypovolemia [20–22]. Overall, pathogenesis of sepsis includes macrovascular and microvascular dysfunction, immunologic and autonomic dysregulation, septic AKI may occur in the absence of renal hypoperfusion and hemodynamic derangement and even in the presence of normal or increased global renal blood flow [5,23–25]. Premature cell senescence plays a critical role in septic AKI. Study by Chaojin Chen and colleagues indicated that LXA4 exerted its renoprotective effects by blocking the crosstalk between inflammation and premature senescence in a PPAR-dependent manner [26]. It is different from the major causes of nonseptic AKI which are renal hypoperfusion and associated ischemia in the critically ill patients [7,27,28]. This study shows sepsis increase excess mortality compared to nonseptic pathogenic factors such as trauma and cardiac insufficiency, etc in matched clinical conditions.

Sepsis may have specific prognostic implications when it compared with other AKI causes. There was study showed an overall mortality of 27% in post-traumatic AKI was comparable with what had been observed in a general ICU population [29]. Emergency surgery increased the risk of postoperative sepsis over 6-fold after regarding the confounders in aged patients [30]. Some studies in patients developing AKI after cardiac surgery showed short-term mortality of cardiac surgery-associated AKI (CSA-AKI) was reported between 15–30% [31]. Clinical and prognostic relevance of AKI in the setting of ST-elevation acute myocardial infarction (STEMI) complicated by cardiogenic shock (CS) was evaluated by study of Marenzi G, et al. [32]. Ninety-seven consecutive STEMI patients with CS were included and 52 (55%) patients developed AKI. Patients developing AKI had a high mortality rate of 50 percentage points. In the BEST Kidney trial analysis [16], septic AKI were 50% higher of the odds of dying in hospital in compared with non-septic AKI. Visibly, the composition of the non-septic group and its proportion of conditions with poor prognosis (such as CS) greatly influence the different prognosis between septic and nonseptic AKI.

The various mortalities of septic AKI were reported in different studies. Our study showed a hospital mortality of 33.1% in matched AKI patients with sepsis compared with 24.0% of their matched AKI controls without sepsis. Angus et al. examined 192,980 patients with severe sepsis from seven US states [33]. They found AKI occurred in 22% and was associated with a mortality of 38.2%. The SOAP cohort study recruited 3147 patients [34]. Of them, AKI occurred in 51% of sepsis cases and was related to an ICU mortality of 41%. Different from studies above, this study highlights the attributable mortality of sepsis for AKI, and a close link was observed between increased mortality and sepsis severity. There are a lot of studies focused on septic AKI, we should also be concerned for the AKI patients who developed sepsis after AKI. Whether sepsis developed before, simultaneously or after AKI, it would deteriorate organ function and contribute excess mortality for AKI. Furthermore, septic shock contributed to major excess mortality for AKI than sepsis. The seriousness of this condition emphasized the need for prompt and appropriate intervention. Prevention of sepsis development and progress to septic shock may represent a potential key therapeutic target for AKI and decrease mortality.

This study still has some limitations. The database was prospectively collected and detailed clinical data allowed us to construct careful sequential matching. However, the study was analyzed retrospective, which may make some hidden biases. Secondly, we studied AKI and sepsis in the first 5 days after ICU admission, there were still a minority of patients develop AKI and/or sepsis after 5 days, this may slightly affact the results of excess mortality attributable to sepsis for AKI patients. Nevertheless, development of AKI and sepsis after day 5 is uncommon [18]. Furthermore, the majority of AKI and sepsis were diagnosed in the first 2 days in this study.

## Conclusion

The attributable hospital mortality of total sepsis for AKI were 9.1% (95% CI 4.8-13.3%). Septic shock contributes to more excess mortality rate for AKI than sepsis.

## Supplementary Material

Supplemental Material

## Data Availability

Data not available due to ethical restrictions.

## Geolocation information

Beijing, China.
