# Supplementary material for: The attributable mortality of sepsis for acute kidney injury: a propensity-matched analysis based on multicenter prospective cohort study
Source: Ren Fail. 2023 Jan 13;45(1):2162415. doi: 10.1080/0886022X.2022.2162415 (PMC9848315; doi:10.1080/0886022X.2022.2162415)
Supplement: Supplemental Material [file IRNF_A_2162415_SM6056.pdf]

Data Supplement

The list of 18 trial sites of 16 hospitals in database

Department of Critical Care Medicine, Fuxing Hospital, Capital Medical University, Beijing, China；Department of Critical Care Medicine, West China Hospital, Sichuan University, Sichuan, China；Medical Intensive Care Unit, Peking Union Medical College Hospital, Beijing, China；Department of Critical Care Medi cine, Guangdong Geriatric Institute, Guangdong General Hospital, Guangdong, China；Department of Critical Care Medicine, The First Affiliated Hospital of China Medical University, Shenyang, China; Surgical Intensive Care Unit, Department of Anesthesiology, ZhongShan Hospital, FuDan University, Shanghai, China; Intensive Care Unit, The First Hospital of Jilin University, Changchun, China; Department of Critical Care Medicine, China-Japan Friendship Hospital, Beijing, China; Department of Critical Care Medicine, Beijing Friendship Hospital, Capital Medical University, Beijing China; Surgical Intensive Care Unit, Beijing Chaoyang Hospital, Capital Medical University, Beijing, China; Department of Respiratory and Critical Care Medicine, Beijing Institute of Respiratory Medicine, Beijing Chaoyang Hospital, Capital Medical University, Beijing, China; Department of Critical Care Medicine, General Hospital of Ningxia Medical University, Ningxia, China; Department of Critical Care Medicine, Xiangya Hospital, Central South University, Changsha, China; Department of Critical Care Medicine, Beijing Tongren Hospital, Capital Medical University, Beijing, China; Department of Critical Care Medicine, Peking University Third Hospital, Beijing,

China; Surgical Intensive Care Unit, Xuanwu Hospital, Capital Medical University, Beijing, China; Department of Critical Care Medicine, Beijing Tiantan Hospital, Capital Medical University, Beijing, China.

Supplemental Table 1. Characteristics of matched AKI patients with sepsis and their controls without sepsis in five separate time points

DAY 1

Variables	Matched AKI with sepsis (n = 224)	Matched AKI without sepsis (n = 224)	*p* value	Standardized Difference
Age, years	71 (58, 80)	70 (55, 80)	0.800	-0.080
Male gender	142 (63.4)	133 (59.4)	0.438	0.082
BMI, kg/m^2	19.0 (17.6, 20.6)	19.1 (17.1, 20.8)	0.599	0.021
COPD/asthma	19 (8.5)	15 (6.7)	0.593	0.000
Cardiovascular disease	53 (23.7)	46 (20.5)	0.495	-0.010
Chronic liver disease	4 (1.8)	4 (1.8)	1.000	0.005
Cancer	19 (8.5)	23 (10.3)	0.627	-0.035
Diabetes	63 (28.1)	59 (26.3)	0.750	-0.082
Hypertension	103 (46.0)	100 (44.6)	0.849	0.004
CKD	28 (12.5)	35 (15.6)	0.415	0.078
AKI stage 2-3, day 1	154 (68.8)	151 (67.4)	0.852	-0.034
Nonrenal SOFA, day 1	5 (2, 8)	5 (3, 8)	0.568	0.092
Mechanical ventilation	154 (68.8)	154 (68.8)	1.000	0.002

Values are median (interquartile range) or *n* (%), *BMI* body mass index, *COPD* chronic obstructive pulmonary disease, *CKD* chronic kidney disease, *AKI* acute kidney injury, *SOFA* sequential organ failure assessment

DAY 2

Variables	Matched AKI with sepsis (n = 186)	Matched AKI without sepsis (n = 186)	*p* value	Standardized Difference
Age, years	62 (48, 76)	62 (47, 75)	0.626	0.008
Male gender	118 (63.4)	122 (65.6)	0.745	-0.010

BMI, kg/m^2	19.4 (17.6, 21.4)	19.8 (17.8, 21.3)	0.512	0.032
COPD/asthma	10 (5.4)	5 (2.7)	0.292	-0.092
Cardiovascular disease	38 (20.4)	31 (16.7)	0.424	0.001
Chronic liver disease	5(2.7)	5 (2.7)	1.000	-0.004
Cancer	13(7.0)	16 (8.6)	0.700	0.003
Diabetes	45 (24.2)	40 (21.5)	0.622	0.024
Hypertension	70 (37.6)	72 (38.7)	0.915	0.036
CKD	21(11.3)	17 (9.1)	0.608	0.015
AKI stage 2-3, day 2	112 (60.2)	101 (54.3)	0.268	0.012
Nonrenal SOFA, day 2	4 (2, 7)	4 (2, 7)	0.756	0.087
Mechanical ventilation	136 (73.1)	133 (71.5)	0.817	-0.134

Values are median (interquartile range) or *n* (%), *BMI* body mass index, *COPD* chronic obstructive pulmonary disease, *CKD* chronic kidney disease, *AKI* acute kidney injury, *SOFA* sequential organ failure assessment

DAY 3

Variables	Matched AKI with sepsis (n = 136)	Matched AKI without sepsis (n = 136)	*p* value	Standardized Difference
Age, years	70 (54, 82)	68 (51, 82)	0.711	0.067
Male gender	90 (66.2)	88 (64.7)	0.899	-0.032
BMI, kg/m^2	19.3 (18.2, 21.2)	18.7 (17.8, 20.1)	0.166	0.094
COPD/asthma	9 (6.6)	6 (4.4)	0.597	0.069
Cardiovascular disease	29 (21.3)	29 (21.3)	1.000	-0.004
Chronic liver disease	3 (2.2)	5 (3.7)	0.722	-0.037
Cancer	11 (8.1)	14 (10.3)	0.675	0.008
Diabetes	31 (22.8)	28 (20.6)	0.769	-0.012
Hypertension	56 (41.2)	56 (41.2)	1.000	-0.002
CKD	10 (7.4)	9 (6.6)	1.000	0.003
AKI stage 2-3, day 3	44 (32.4)	38 (27.9)	0.657	-0.028
Nonrenal SOFA, day 3	6 (4, 8)	6 (3, 8)	0.669	0.040
Mechanical ventilation	101 (74.3)	108 (79.4)	0.389	0.065

Values are median (interquartile range) or *n* (%), *BMI* body mass index, *COPD* chronic obstructive pulmonary disease, *CKD* chronic kidney disease, *AKI* acute kidney injury, *SOFA* sequential organ failure assessment

DAY 4

Variables	Matched AKI with sepsis (n = 45)	Matched AKI without sepsis (n = 45)	*p* value	Standardized Difference
Age, years	68 (58, 78)	68 (55, 74)	0.786	0.028

Male gender	30 (66.7)	23 (51.1)	0.198	0.090
BMI, kg/m^2	19.1 (17.8, 21.4)	19.2 (16.8, 21.4)	0.540	0.124
COPD/asthma	1 (2.2)	1 (2.2)	1.000	0.000
Cardiovascular disease	10 (22.2)	7 (15.6)	0.591	-0.006
Chronic liver disease	1 (2.2)	0 (0.0)	1.000	0.000
Cancer	2 (5.9)	6 (11.8)	0.266	-0.035
Diabetes	8 (17.8)	5 (11.1)	0.550	0.002
Hypertension	19 (42.2)	18 (40.0)	1.000	0.000
CKD	6 (13.3)	2 (4.4)	0.266	0.078
AKI stage 2-3, day 4	16 (35.6)	8 (17.8)	0.094	0.095
Nonrenal SOFA, day 4	4 (2, 8)	4 (2, 7)	0.812	-0.010
Mechanical ventilation	30 (66.7)	35 (78.8)	0.347	0.013

Values are median (interquartile range) or *n* (%), *BMI* body mass index, *COPD* chronic obstructive pulmonary disease, *CKD* chronic kidney disease, *AKI* acute kidney injury, *SOFA* sequential organ failure assessment

DAY 5

Variables	Matched AKI with sepsis (n = 28)	Matched AKI without sepsis (n = 28)	*p* value	Standardized Difference
Age, years	79 (60, 83)	77 (63, 83)	0.889	0.068
Male gender	20 (71.4)	19 (67.9)	1.000	0.000
BMI, kg/m^2	19.0 (17.3, 20.9)	19.5 (17.4, 20.6)	0.517	0.075
COPD/asthma	5 (17.9)	6 (21.4)	1.000	-0.138
Cardiovascular disease	6 (21.4)	4 (14.3)	0.729	-0.004
Chronic liver disease	0 (0.0)	0 (0.0)	-	-
Cancer	3 (10.7)	0 (0.0)	0.236	0.028
Diabetes	7 (25.0)	6 (21.4)	1.000	0.000
Hypertension	13 (46.4)	13 (46.4)	1.000	0.000
CKD	4 (14.3)	2 (7.1)	0.669	-0.014
AKI stage 2-3, day 5	5 (20.0)	7 (25.0)	0.386	0.036
Nonrenal SOFA, day 5	4 (2, 6)	4 (2, 5)	0.849	0.204
Mechanical ventilation	23 (82.1)	24 (85.7)	1.000	0.000

Values are median (interquartile range) or *n* (%), *BMI* body mass index, *COPD* chronic obstructive pulmonary disease, *CKD* chronic kidney disease, *AKI* acute kidney injury, *SOFA* sequential organ failure assessment
